# Maternal and paternal psychological control and adolescents’ negative adjustment: A dyadic longitudinal study in three countries

**DOI:** 10.1371/journal.pone.0251437

**Published:** 2021-05-14

**Authors:** Emanuele Basili, Antonio Zuffianò, Concetta Pastorelli, Eriona Thartori, Carolina Lunetti, Ainzara Favini, Flavia Cirimele, Laura Di Giunta, Maria Gerbino, Dario Bacchini, Liliana Maria Uribe Tirado, Jennifer E. Lansford

**Affiliations:** 1 Department of Psychology, Sapienza University of Rome, Rome, Italy; 2 Department of Human Studies, University of Naples “Federico II”, Naples, Italy; 3 Facultad de Psicología, Universidad de San Buenaventura, Medellin, Colombia; 4 Center for Child and Family Policy, Duke University, Durham, North Carolina, United States of America; Liverpool John Moores University, UNITED KINGDOM

## Abstract

Psychological Control (PC) interferes with autonomy-related processes in adolescence and has a negative impact on adolescents’ development related to internalizing and externalizing problems. Several scholars suggested that PC can be used differently by mothers and fathers. However, these differences are still understudied and mainly grounded on maternal and/or adolescents’ perspectives, leading to potentially incomplete inferences on the effects of PC. The present study extends previous research on PC in two directions. First, we tested the dyadic and cumulative effects of maternal and paternal PC on adolescents’ antisocial behaviors and anxious-depressive symptoms. Secondly, we explored the cross-cultural generalizability of these associations in three countries: Italy, Colombia, and USA. Participants included 376 families with data from three consecutive years (T1, adolescents’ age = 13.70). Mothers’ and fathers’ reports of PC and youth’s reports of antisocial and internalizing behaviors were assessed. Using the Actor-Partner Interdependence Model (APIM) we found that maternal PC predicted adolescents’ reported antisocial behaviors whereas paternal PC predicted lower anxious-depressed symptoms. Comparisons across countries evidenced the cross-cultural invariance of the longitudinal APIM across Italy, Colombia, and USA. The practical implications of these results are discussed.

## Introduction

Psychological Control (PC) is defined as a pattern of behaviors through which parents try to intrude in the psychological world of the adolescent by using manipulative strategies such as guilt induction, verbal constraint, and love withdrawal [1]. PC is distinct from behavioral control, which involves the communication of clear rules and consistent expectations for appropriate behavior and efforts to monitor the child’s behaviors and activities (e.g. [2, 3]).

A number of studies have found PC to be especially detrimental for adolescents’ development [4–6]. Moreover, research has highlighted the relevance of PC in the dynamics of family systems and subsystems (i.e., dyads), for which constructs such as enmeshment, intrusiveness, lack of acknowledgment, and boundary violation are pivotal (e.g., [7–9]). Yet, although studies on mother-child dyads are increasing, there has been limited examination on the dyadic interplay in the use of PC through the perspective of both parents and focusing on their specific parental couple dynamic [10]. Furthermore, because the role of fathers has often been omitted or neglected in PC studies [11], there is very limited evidence on the different (or similar) roles mothers and fathers play when using manipulative and intrusive parental strategies with their children. The use of PC has been demonstrated to be also culturally relevant, and the debate about whether the link between PC and adolescents’ functioning is similar across cultures is still ongoing [12]. Even if researchers are generally confident in claiming the cross-cultural equivalence of psychologically controlling strategies [6, 13], these hypotheses have not been tested considering mothers’ and fathers’ dyadic processes.

The current study directly examined the reciprocal associations between parental PC dyadic functioning for both mothers and fathers over three years and across three different cultural sites—Italy, USA, and Colombia. We also tested the relations of these longitudinal dyadic associations with adolescents’ internalizing and externalizing behaviors.

### Dyadic contributions of maternal and paternal psychological control to adolescents’ negative outcomes

Parental behaviors within two-parent families are highly correlated [14, 15]. Although scholars suggested that maternal and paternal PC might be conceptually and empirically distinguishable, they are nonetheless theorized to influence one another in a reciprocal way [10, 11]. Consistent with Family Systems Theory [9]—which posits family members are interconnected and each person within a family plays a precise role in relation to the other members and the family as a whole—individuals within the same dyad makes likely that one’s experience of parenting across time is linked not only to one’s own but also to the partner’s parenting (i.e. *partner effects*; [15]).

However, little is known about the mutual and dyadic influence of maternal and paternal PC on adolescents’ well-being. Specifically, research has failed to include both mothers and fathers and control for their reciprocal influences when exploring the longitudinal relation between PC and adolescents’ adjustment (e.g. [13, 16]). Although an increasing number of studies have focused on associations within mother-adolescent dyads (e.g. [17]), very few studies addressed the question of a reciprocal association between maternal and paternal PC over time. For instance, one study used the Actor–Partner Interdependence Model (APIM) to test the longitudinal dyadic associations of maternal and paternal PC over two years [18]. Results evidenced significant reciprocal associations (i.e., partner effects) showing the mutual influences of mothers’ and fathers’ use of PC over time. Similarly, another study directly tested maternal and paternal reciprocal associations in mothers’ and fathers’ daily reports of PC [19]. The authors, using multilevel modeling, also investigated the possibility that mothers’ emotions could be related to fathers’ daily use of PC beyond fathers’ own emotions (and vice versa). Although the results showed that there were no statistically significant cross-parent effects, the authors introduced the relevance of testing these dyadic dynamics to interpret the effects on offspring’s adjustment.

In the current literature, it is unclear–according to a developmental family perspective—whether the longitudinal association between maternal and paternal PC and adolescents’ adjustment is due to the overlapping variance shared between the two parents at an earlier time point or to the unique role that each partner plays in predicting adolescents’ adjustment at subsequent times. The one study to note following this direction tested the independent and joint effects of mothers’ and fathers’ autonomy-relevant parenting, including PC, during early and middle adolescence (13 to 17 years old; [20]). Results showed that —when considering both mothers and fathers and testing joint and interactive effects of both parents’ PC on adolescents’ well-being —fathers’ but not mothers’ PC was a unique predictor of adolescents’ externalizing and internalizing problems.

Within the family environment, parents might influence each other in their use of PC through a process of co-parental negotiation (e.g., [21]). This pattern of influence might be maintained across time and determine different outcomes for adolescents’ adjustment [22, 23].

### Psychological control and adolescents’ internalizing and externalizing behaviors: Differences and similarities by parents’ and adolescents’ gender

PC is especially problematic during adolescence because it interferes with autonomy development and promotes continued dependence on parents (e.g., [6]). Both cross-sectional and longitudinal data showed that PC has been consistently associated with negative outcomes such as adolescents’ anxiety (e.g., [24]), depression (e.g., [13]), delinquency [25], and antisocial behavior (e.g., [26]). Generally, mothers have been considered as the primary caregivers of their children (e.g., [27]), but recent empirical evidence found that both parents may contribute to the degree to which their adolescent children display externalizing and/or internalizing behaviors [28]. Regarding externalizing behavior, cross-sectional research showed that youth-reported maternal PC has a stronger association with antisocial behavior than paternal PC (e.g., [29]). Longitudinally, maternal and paternal PC, as reported by both parents and adolescents, has been shown to be related to relational aggression, although bidirectional associations were found only for mother- and not for father-child dyads [30]. Murray et al. [31] found that youth-reported paternal PC was associated, over 2 years, with higher levels of aggression among youth who reported a low-quality relationship with their mother.

Regarding internalizing problems, cross-sectional data showed that adolescent reports of their parents’ PC predicted more internalizing problems, such as depressive symptoms [32] and lower self-esteem [33]. Longitudinally, Rogers et al. [34] examined the developmental course of adolescents’ perceptions of mothers’ and fathers’ PC across an 8-year period spanning early to late adolescence (ages 12–19). They found that mothers’ (but not fathers’) depressive symptoms, reported by the youth at age 12, predicted higher levels of youth’s perceived PC and more problematic growth in depressive and anxiety symptoms across adolescence. Also, longitudinally, adolescents’ perceived paternal PC has been shown to lead to both externalizing and internalizing behaviors [20]. In contrast, other studies did not find gender differences in levels of maternal and paternal PC and their effects on adolescents’ negative adjustment [11, 35–37]. However, these findings were mostly based on the adolescents’ perceptions of their parents’ PC and failed to include both mothers’ and fathers’ perspectives on youth adjustment. Research that focused on parents’ self-reports is scarcer and provided mixed findings on the contribution of mothers’ and fathers’ PC to adolescents’ internalizing and externalizing behaviors [31]. In order to better examine the quality of interactions within families, further longitudinal research on parental behaviors and child outcomes in mother—father dyads are needed (e.g. [20]). Although fathers tend to have more distant relationships with their children compared with mothers [38], mothers and fathers tend to show similar parenting behaviors both according to their own reports and child reports [39]. The degree of behavioral similarity among co-parents is relatively high: maternal and paternal levels of PC are moderately positively correlated, even when children or parents are asked to report these parenting behaviors [18].

Additionally, the influence of PC on problematic outcomes among youth has been found to vary by adolescent gender. However, these findings are often inconsistent, as some findings suggest, for instance, that PC is more problematic among girls [16, 40], whereas other findings suggest that it is more problematic for adolescent boys [32, 41]. Moreover, higher levels of perceived maternal PC, but not paternal PC, were associated with greater anxiety in girls, but not in boys [42]. Similarly, early female adolescents, but not male adolescents, whose mothers were high in PC during middle childhood and adolescence, were found to have higher levels of anxiety/depression and antisocial behaviors [25]. Similarly, male adolescents perceive their mothers as using more PC than their fathers [32]. Nevertheless, other studies found no significant differences between maternal and paternal PC and adolescents’ gender, consistent with empirical findings and theory suggesting that PC interferes with the fundamental need for autonomy and independence similarly for adolescent males and females (e.g., [6, 43]).

In summary, despite extensive research on PC and adolescents’ adjustment, several inconsistencies have emerged in previous studies. Specifically, few studies examined the effect of parental PC on both internalizing and externalizing behaviors (e.g., [28]), and the majority of studies were based on adolescents’ reports (i.e., [44]) or on parents’ reports only, even when data were available from both parents and adolescents (e.g., [10]).

#### PC in a cross-cultural framework

There are important influences on fathers and mothers depending on their cultural background and nationality [27, 45], and parenting practices are suited to the belief systems and the cultural and community context in which they are embedded. In two-parent families that endorse clear division and gender-based roles in parenting, mothers and fathers may attend to, or respond to, different kinds of contextual and cultural influences. Accordingly, PC-related processes have been demonstrated to be also culturally relevant, and the debate about whether the link between PC and adolescents’ functioning is similar across cultures [13, 32] is still ongoing. According to the Self-determination Theory, those parenting behaviors that are perceived as controlling undermine the innate and universal needs of autonomy, competence, and relatedness (e.g., [6, 46]). Several studies that focused on the culture-specific perspective, showed that parental control may be perceived as more normative in non-Western and Asian cultures [47, 48] and as such may be unrelated to adolescents’ adjustment. For example, Chao & Aque [49] reported that PC was related to greater anger, conduct problems, and drug use among European- American, but not among Chinese- American 14 years old adolescents, though the effects on depression and anxiety symptoms were similar.

Adolescents from different countries and cultures may provide different meanings and, consequently, perceive differently the level and quality of parental control, for example evaluating it as less negative and less intrusive. This aspect has been mostly investigated in Eastern and Asian cultures where parental control is considered more normative (e.g., [50]). Similarly, different hypotheses were formulated on individualistic vs collectivist cultures. Adolescents growing up in an individualistic culture that stresses the importance of autonomy and personal independence might be influenced more negatively by psychologically controlling parenting [1, 10].

In the context of the traditional values of respect and familism, for example, Halgunseth et al. [51] hypothesized that the use of control related behaviors among Latinx families may reflect the specific cultural goals to maintain these values. Similarly, among the Mediterranean cultures, Italian mothers and fathers of 16 to 21 years old adolescents have been found to value interdependence and family loyalty, assigning different meaning to controlling parenting [52, 53] compared to Northern Europe and the US [54]. Regarding differences related to parental gender, Julian et al. [55] found that Hispanic fathers reported more emphasis on child control of emotions (i.e. not crying, hiding anger) than European American mothers or fathers and described Mexican-American mothers as using more guilt inducing strategies than European American mothers. Fathers with less traditional gender roles from both Eastern and Western societies tend to be more involved with their children [56, 57], and the effects of paternal psychologically controlling strategies on youth’s outcomes might be heavily influenced by values as *machismo*, especially for Latinx youth [58].

However, studies endorsing the universality perspective have shown that the relation between perceived PC and adolescents’ adjustment is similar, for instance, across American and European cultures (e.g., [30, 59]), although it may manifest in different domains of adolescents’ behavior and functioning. Chen and colleagues [60], for example, examining how controlling parenting practices are perceived and dealt with, found that, once the situations were perceived as controlling, adolescents from Belgium and China (13–19 years old) suffered to a similar degree in terms of need frustration. Similarly, Furukawa et al. [61] compared shame- and guilt- proneness in Japanese, Korean, and US children (grades 3rd–6th), and found similar patterns of correlations in the associations with aggression-relevant constructs and functional behaviors towards failures and transgressions. Barber et al. [13] compared parents and adolescents (13–19 years old) from five cultures–Thailand, Costa Rica, South Africa (three racial groups)—and found that psychologically controlling parenting was associated with adolescents’ depression and antisocial behaviors across all cultural groups. Similarly, McNeely and Barber [62] asked adolescents from 12 cultural groups in Africa, Asia, Australia, North and South America, Europe, and the Middle East (adolescents’ age range 14–17) to define the term PC and found cross-cultural consensus in the participants’ response. Findings regarding how maternal and paternal PC are associated with adolescents’ adjustment problems in samples embedded in different cultures are still needed.

### The present study

**Fig 1:**
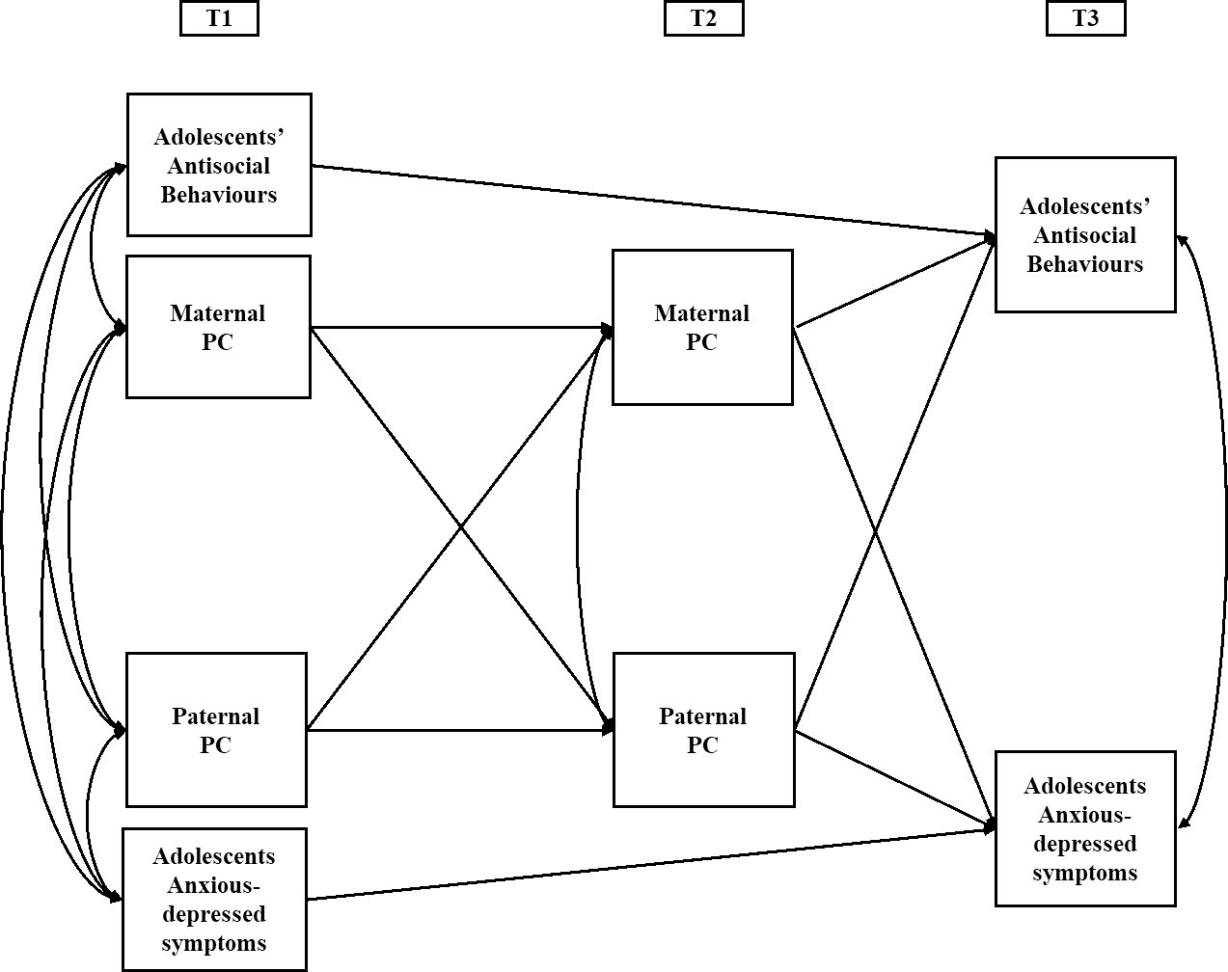
Conceptual APIM model of maternal and paternal PC.

In the present study we used three waves of data from Italian, U.S. American, and Colombian families to investigate three main aims. First, we tested the longitudinal dyadic associations between maternal and paternal PC and their association with adolescents’ adjustment one year later. Specifically, we examined longitudinal associations between maternal and paternal use of PC using the APIM to account for the interdependence between partners and to test for the direct effects of one’s own parenting over time (i.e. *actor effects*) and the direct effects of one partner’s parenting on the other’s (i.e. *partner effects*) [63]. The APIM framework allows also a direct test of the effects of these dyadic associations on the outcome of interest while controlling for stability and autocorrelation effects (Conceptual Model in Fig 1). Although bidirectional relations have been studied between maternal PC and adolescents’ adjustment (e.g., [2, 64]), mothers’ and fathers’ PC over time has rarely been modeled. Findings based only on mothers’ and adolescents’ perceptions led scholars to erroneously presume that the effects of parental strategies would apply equally to all youth and parental configurations (e.g., [65]). This potential risk demands the effects of PC to be examined controlling for contributions of the other parent. Thus, we expected positive and significant *actor* and *partner effects* for both parents in predicting one’s own and one’s partner’s level of PC from one year to the next [18]. Furthermore, we expected PC to be positively associated with both antisocial behaviors and anxiety-depressive symptoms [1, 59]. We also tested for indirect effects of parental PC on adolescents’ adjustment through partners’ level of PC. In a dyadic framework (e.g. Family Systems Theory), it is hypothesized that the effect of one parent’s PC might depend on whether the other parent also engages in the same parental strategy. Thus, indirect actor and partner associations may emerge representing the long-lasting implications of parents’ earlier levels of PC for their own and partners’ PC. Despite the exploratory nature of these analyses, and the absence of prior findings supporting similar pathways for parental PC, this hypothesis was tested in the present study.

Second, we examined whether the hypothesized model was generalizable cross-culturally by using multi-group comparisons among the three countries. To our knowledge, no previous study tested parental actor and partner effects of PC and their associations with adolescents’ adjustment in a cross-cultural framework. However, according to previous studies suggesting how PC-related mechanisms might be considered universal [64], we hypothesized that the proposed model would be tenable for the three countries.

Third, because the literature on gender differences in PC has reached mixed conclusions (e.g., [10]), we explored potential gender differences in the hypothesized effects by testing the moderating role of parental and adolescents’ gender in the dyadic model.

## Materials and methods

### Participants

Participants—recruited from the longitudinal study entitled Parenting Across Cultures (e.g., [66])—included 376 families from Italy (n = 148), Unites States of America (n = 154), and Colombia (n = 74) with data collected at wave 5 (Time 1; T1), wave 6 (Time 2; T2), and wave 7 (Time 3; T3) of the larger study. Adolescents were 13.70 years of age (SD = .67; range = 12–16) at T1, 14.95 years (SD = .73; range = 13–17) at T2, and 15.99 years (SD = .78; range = 14–18) at T3.

The adolescents’ gender distributions, and sample’s demographics by location at T1 were: Rome and Naples, Italy (48.6% female, age = 13.5), Medellín, Colombia (54.1% female, age = 13.4; Durham, North Carolina, United States (44% female, age = 14.02). Adolescent boys and girls were equally distributed among countries (χ^2^ (2) = 1.738, *p* = .419, Cramer’s *V =* .*068*). Mothers reported that 79.3% were married, 11.2% were never married or cohabitating, 1.3% were remarried and 8.1% were separated or divorced. The non-resident parent (if the couple was separated or divorced) also could participate. Mothers averaged 43.02 years (SD = 6.53) and fathers 45.70 years of age (SD = 6.91) at T1. Mothers completed 12.88 years (SD = 4.93) and fathers completed 12.53 years of education (SD = 5.12) on average. Family income was reported using 10 income ranges on an ordinal scale rated from 1 to 10; 21.5% of families reported income in the lowest two categories, and 22.9% reported income in the highest two income categories.

### Procedure

Letters describing the study were sent home with youths, and parents were asked to return a signed form if they were willing to be contacted further. Families were then enrolled in the study and after obtaining approvals from institutional review boards, parental informed consent, and child assent, questionnaires were completed in the participant’s home or location of their choosing (e.g., school). Participants were given the choice to complete measures orally or in writing. Testing sessions lasted approximately two hours. IRB at Sapienza University of Rome approved the study’s protocol and procedure.

### Measures

#### Parental psychological control

At T1 and T2 mothers and fathers completed measures assessing their perceptions of their PC via an adapted version of the Psychological Control and Autonomy Granting Scale [1, 67]. Parents reported their rates of agreement on a 4-point scale with 1 = *“Strongly Disagree”*, to 4 = “*Strongly Agree”*. Items were averaged to create a single dimension of PC including items reflecting parents’ perceptions of their use of PC (8 items, e.g. “I act cold and unfriendly if my child does something I don’t like.”). According to the definition provided by Barber, [1] the scale included parental strategies aimed to evoke feelings of guilt, sadness, and worries in the youth for having done things that have a negative emotional impact on another family member. These strategies may also include the withdrawal of love, parental threat of leaving the interaction with the child when he or she does something contrary to expectations, or the interference with and containment of the child’s expression of opinions and ideas as a way for parents to dominate conversations and invalidate the child’s views. Descriptive statistics and Cronbach alphas by study site are reported in Table 1A–1C.

**Table 1:** a. Descriptives, reliability values and correlations among the examined variables in Italy. b. Descriptives, reliability values and correlations among the examined variables in USA. c. Descriptives, reliability values and correlations among the examined variables in Colombia.

(a)												
	M	SD	α		(1)	(2)	(3)	(4)	(5)	(6)	(7)	(8)
*Psychological Control*												
(1) Mother report T1	2.48	.61	.75	(1)	1							
(2) Father report T1	2.44	.61	.77	(2)	.28**	1						
(3) Mother report T2	2.42	.64	.77	(3)	.69**	.44**	1					
(4) Father report T2	2.30	.63	.79	(4)	.35**	.71**	.46**	1				
*Adolescents’ reported adjustment*												
(5) Antisocial Behavior T1	.17	.17	.61	(5)	.15	.08	.13	.17*	1			
(6) Anxiety-Depression T1	.53	.35	.78	(6)	-.05	-.13	-.09	-.14	.32**	1		
(7) Antisocial Behavior T3	.21	.19	.64	(7)	.11	-.00	.17*	.09	.63**	.21*	1	
(8) Anxiety-Depression T3	.60	.43	.85	(8)	-.15	-.16	-.11	-.27**	.15	.57**	.09	1
(b)												
*Psychological Control*												
(1) Mother report T1	2.11	.54	.71	(1)	1							
(2) Father report T1	2.16	.51	.69	(2)	.20*	1						
(3) Mother report T2	2.13	.58	.76	(3)	.58**	.32**	1					
(4) Father report T2	2.08	.56	.76	(4)	.29**	.55**	.35**	1				
*Adolescents’ reported adjustment*												
(5) Antisocial Behavior T1	.17	.17	.60	(5)	.02	.14	.00	.25**	1			
(6) Anxiety-Depression T1	.40	.36	.82	(6)	-.10	-.01	-.01	.00	.32**	1		
(7) Antisocial Behavior T3	.28	.24	.70	(7)	.03	-.00	.04	-.01	.37**	.23**	1	
(8) Anxiety-Depression T3	.54	.46	.87	(8)	.01	-.16*	-.01	-.10	.01	.51**	.31**	1
(c)												
*Psychological Control*												
(1) Mother report T1	2.86	.52	.70	(1)	1							
(2) Father report T1	2.72	.57	.75	(2)	.20	1						
(3) Mother report T2	2.89	.52	.69	(3)	.61**	.24*	1					
(4) Father report T2	2.68	.61	.80	(4)	.30*	.50**	.11	1				
*Adolescents’ reported adjustment*												
(5) Antisocial Behavior T1	.22	.18	.54	(5)	-.03	.17	.09	.07	1			
(6) Anxiety-Depression T1	.56	.34	.71	(6)	.10	.04	-.01	-.01	.24*	1		
(7) Antisocial Behavior T3	.33	.25	.64	(7)	.07	.20	.27*	.09	.56**	.19	1	
(8) Anxiety-Depression T3	.67	.47	.85	(8)	-.00	.09	.07	-.14	.27*	.53**	.34**	1

Note. *p < 0.05;

**p≤0.01

#### Anxiety/depression and Antisocial behavior

Adolescents’ reports of anxiety/depression and antisocial behavior were assessed using the Youth Self-Report (YSR; [68]). Items were rated on a 3-point scale with 0 = “*Not true*”, 1 = “*Somewhat/Sometimes true*”, 2 = “*Very/Often true*”. The Antisocial behavior scale—11 items, e.g. “I steal things from places other than home”- was used in the current study to index externalizing behavior problems, and the anxiety/depression scale—16 items, e.g. “I worry a lot”—was used to index internalizing problems. Antisocial behavior and anxiety-depression scale scores from the YSR were created for T1 and T3. Descriptive statistics and Cronbach alphas by study site are reported in Table 1A–1C.

#### Control variables

We controlled for parental marital status and number of children in the household. We also controlled for an index of family socioeconomic Status (SES), created by standardizing and averaging parental education and family income (r >.60 in the three countries). Adolescents’ gender (coded 1 for males and 2 for females) was used as a moderator in the multi-group analysis.

### Attrition

As described earlier, parental reports of PC were collected at T1 and T2; youth reported anxious-depressed and antisocial behaviors were collected at T1 and T3. The family members’ participation rate remained high across time. Specifically, in Italy, the retention rate from T1 to T2 was 97.95% for mothers and 89.61% for fathers, and the retention rate from T1 to T3 was 95.23% for youth. In the USA the retention rate from T1 to T2 was 92.20% for mothers and 78,57% for fathers, and for youth, the retention rate from T1 to T3 was 91.55%. In Colombia, the retention rate from T1 to T2 was 91.89% for mothers and 85.13% for fathers, and for youth, the retention rate from T1 to T3 was 89.18%. Site-specific sample sizes over time are reported in Table 1. Attrition was principally due to two main reasons: unavailability of the subjects to participate in the later data collections in the ongoing longitudinal study or their unwillingness to participate in that specific wave.

Analysis of variance showed that the missing participants in the three countries did not significantly differ from their counterparts in their marital status, SES, perceived PC, antisocial behaviors, and internalizing symptoms (Cohen’s ds < .15, ps > .70), with a few exceptions. Specifically, in Italy missing fathers reported using less PC F(1,147) = 4.80, p = 0.030; Cohen’s d = .18) compared to their counterparts. Moreover, in the USA missing families had slightly lower SES than the families that continued in the study F(1,152) = 5.12, p = 0.025; Cohen’s d = .18).

### Data analytic approach

The models were tested via M*Plus* 7 [69] by conducting a longitudinal cross-lagged APIM to simultaneously estimate dyadic associations between parental PC across time, while also testing the associations with adolescents’ adjustment [70, 71]. As depicted in Fig 1, we tested a two-waves APIM for mothers’ and fathers’ PC, in which the associations between maternal and paternal PC were tested along with their effect on adolescents’ adjustment one year later. We also controlled for the stability of adolescents’ antisocial behaviors and anxious-depressive symptoms by including their reports at T1. The estimation of the APIM model allows us to account for the interdependence between the two members of the parental couple, by considering the couple as the unit of analysis, to simultaneously estimate both within-partner (i.e., *actor effects*) and cross-partner associations (i.e., *partner effects*) to address more appropriately the directionality of the associations.

First, a model without covariates was used to examine the longitudinal associations across countries (see Fig 1). Then, we tested a covariate-controlled model to test whether associations between the variables were influenced by control variables (i.e. family *SES*, *marital status*, and the *number of children in the household*). Finally, we implemented multi-group path analysis models to test whether there were significant differences in the structural parameters across the three countries and adolescents’ gender. We compared a model in which paths were free to vary across countries to one in which paths were fixed to be equal across sites. If constraining all the paths to be equal yielded a worse fit to the data, using modification indices, site-specific paths were iteratively freed until the optimal model fit was achieved.

We used the robust maximum likelihood estimation method in Mplus. Model adequacy was evaluated using multiple indices [72] including the chi-square (χ^2^) statistics, Comparative Fit Index (CFI) and Root Mean Square Error of Approximation (RMSEA) and Standardized Root Mean Square Residual (SRMR). Models with nonsignificant χ^2^ values, CFI > .90, RMSEA values < .08, and SRMR< .06 were considered to have an acceptable fit. However, given its sensitivity to sample size, a significant χ^2^ should be expected for most models [73]. Alternative models were tested using Δχ^2^ and using also the Akaike information criterion (AIC). Specifically, we considered the ΔAIC comparing the AIC of the hypothesized model with the AIC of the alternative models [74]. According to Burnham and Anderson [74], models with ΔAIC ≤ 2 have substantial support, models with 4 ≤ ΔAIC ≤ 7 have considerably less support, and models with ΔAIC ≥ 10 have essentially no support. Missing values were handled via full information maximum likelihood (FIML; [75]).

Once the best-fitting model was achieved, indirect effects were also examined. Bias-corrected bootstrapping methodology was employed using M*Plus* with 5,000 bootstrap samples [76]. Bootstrapping estimates indirect effects through empirical sampling distributions by calculating confidence intervals. If zero is not included within the intervals, statistical significance is examined, and the null hypothesis of no indirect effects is rejected [77].

## Results

Descriptive statistics, scales’ reliabilities, and Pearson’s correlations among the study variables are presented in Table 1A–1C. All measures had adequate reliabilities. Mothers and fathers showed moderate positive correlations in their use of PC over time in Italy and the USA; these correlations were marginally significant for Colombian parents. Furthermore, Italian and Colombian mothers’ PC was positively associated with higher T3 adolescents’ antisocial behaviors, while a negative significant association was found between Italian fathers’ reports of PC and adolescents’ anxious-depressed symptoms. Both antisocial behaviors and anxious-depressed symptoms showed significant stability in the three countries from T1 to T2.

**Fig 2:**
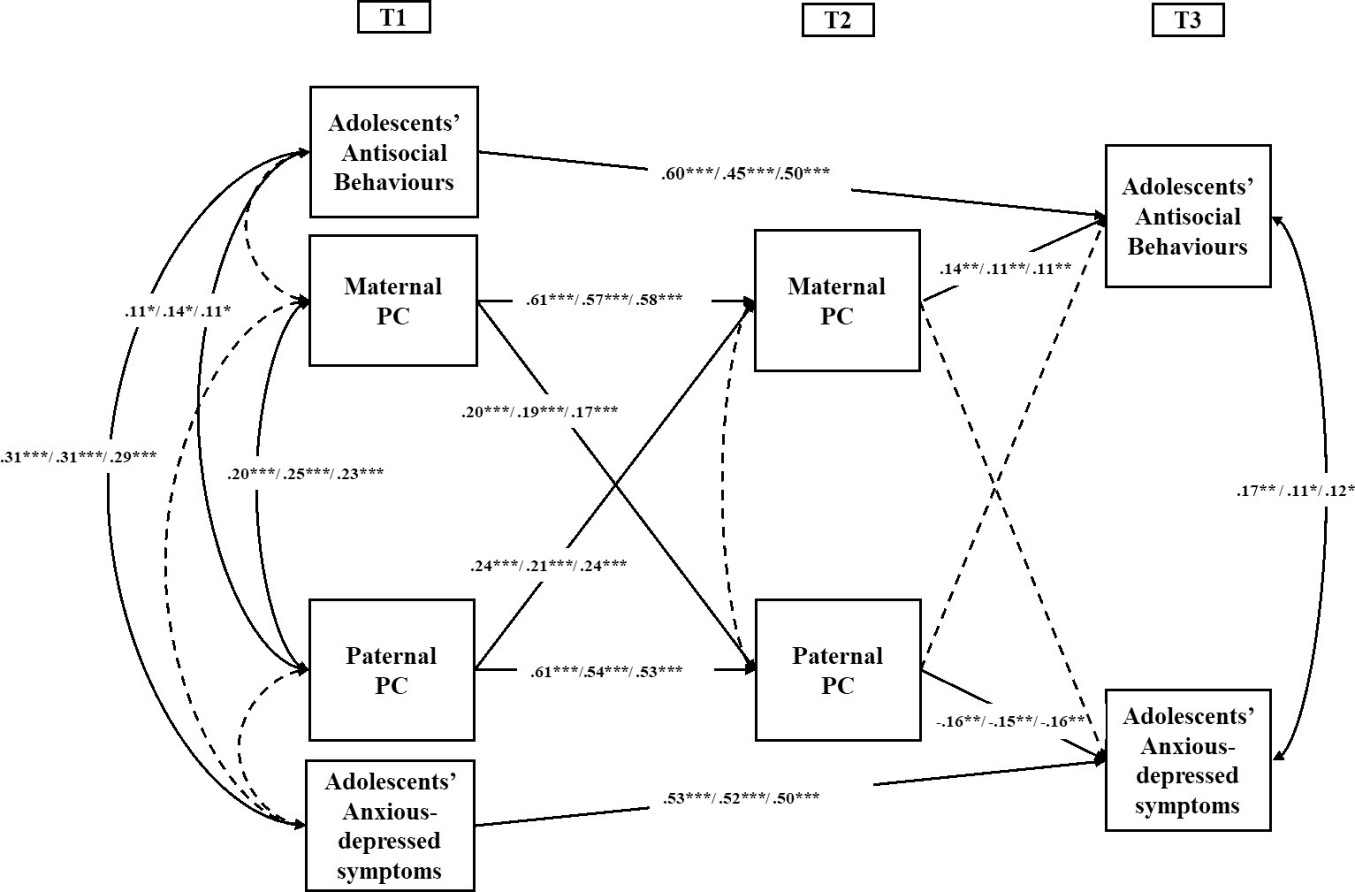
APIM model of relations of maternal and paternal PC and adolescents’(mal)adjustment. * = p < .05; ** = p < .01; *** p < .001. Standardized coefficients are presented for Italy/USA/ Colombia. Dotted lines indicate non- significant associations.

To address the first aim, we fit a model with no covariates and with stability coefficients from T1 to T3 youth-reported *anxiety/depression* and *antisocial behavior* and paths from T1 maternal and paternal *PC* to maternal and paternal PC at T2 (*actor* and *partner* effects) and youth-reported *anxiety/depression* and *antisocial behavior* at T3 (Fig 2). According to the APIM implementation, actor and partner effects were tested simultaneously and the same observed variables relative to parental PC at T1 and T2 were allowed to be error-correlated [63].

The no-covariate model provided a good fit to the data, χ^2^ (10) = 9.189, p = .51, CFI = 1.00, RMSEA = .00, 90%CI = .000 -.053, SRMR = .018, AIC = 2295.70. As shown in Fig 2, the APIM showed that for both mothers and fathers, earlier use of PC predicted their PC one year later (*actor effects*); from T1 to T2, mothers’ and fathers’ earlier use of PC was positively associated with their partner’s later use of PC (*partner effects*). In addition, direct positive significant associations were found between maternal PC and adolescents’ *antisocial behaviors*, while a negative and significant association was found between paternal PC and adolescents’ *anxious-depressive symptoms*. Youth-reported *anxiety/depression* and *antisocial behavior* were moderately stable from T1 to T3.

To exclude the possible influence of the demographic variables in the mentioned associations, we fitted a covariate- controlled model by including T1 *family SES*, *number of children in the household*, and *parental marital status* as covariates to the model with direct paths to all variables in the model and covariances between them. The covariate- controlled model fit the data well, χ^2^ (16) = 16.628, p < .41, CFI = .99, RMSEA = .010, 90%CI = .000–.050, SRMR = .025, AIC = 2428.61. All structural paths depicted in Fig 2 were still significant at the .05 level when controlling for *family SES*, the *number of children in the household*, and *parental marital status*, and no structural differences were found for model associations. Furthermore, the information deriving from the ΔAIC (ΔAIC = 132.90) indicated that, despite the similarity of models’ fit, the alternative covariate-controlled model was a model with less support (ΔAIC ≥ 10) compared to the hypothesized no-covariate model. We then retained the model with no covariates (Fig 2) as the more parsimonious model and proceeded to test it through a multi-group analysis comparing Italy, USA, and Colombia.

### Multiple-group APIM by country

To address the second aim of the study, we tested whether the hypothesized model was generalizable cross-culturally by using multi-group comparisons among the three countries. We first fitted a model in which paths were allowed to vary across countries, and it showed a good fit to the data, χ^2^(30) = 34.924, p = .24, CFI = 0.99, RMSEA = .036, 90% CI = .00, .080, SRMR = .038, AIC = 2344.60. Then, to test cross-cultural invariance, we constrained all the paths to be equal across the three countries. The chi square comparison did not show a significant difference between the unconstrained and the fully constrained model, Δχ^2^(36) = 41,337, p = .15, and the ΔAIC = 30.338 suggested to retain this latter model to examine our results. The final APIM model showed that several longitudinal associations between parental PC and adolescents’ adjustment were invariant across the three countries. The fully constrained model in Fig 2 showed significant and positive *actor* and *partner* effects for both mothers and fathers, meaning that both parents influence each other in the use of PC over time. Moreover, controlling for dyadic associations between mothers and fathers, a direct positive significant association was found between maternal PC and adolescents’ antisocial behaviors, while a negative and significant association was found between paternal PC and adolescents’ anxious-depressive symptoms. No direct and significant associations were found between mothers’ PC and youth’s anxious-depressive symptoms or fathers’ PC and youths’ antisocial behaviors. These associations were found while controlling for the previous levels of adolescents’ adjustment at T1.

In addition to the direct associations (e.g., T1 maternal/paternal PC→T2 maternal/paternal PC→T3 adolescent adjustment), bias-corrected bootstrapping analyses indicated one longitudinal significant indirect associations linking paternal PC to adolescents’ antisocial behaviors via T2 maternal PC (b = .012, SE = .004, 95% CI [.004, .021], β = .036; b = .012, SE = .004, 95% CI [.004, .021], β = .024; b = .012, SE = .004, 95% CI [.004, .021], β = .028; for Italy, USA and Colombia respectively).

### Multiple-group APIM by parents’ and adolescents’ gender

Finally, to test for parental gender differences in each country, we compared models in which maternal and paternal actor and partner effects were allowed to vary (unconstrained model) against models in which these paths were constrained to be equal (constrained model). Chi square comparisons provided no significant differences between mothers and fathers, suggesting that parental stability and reciprocal influences were equivalent across parents (See S1 Table).

Finally, we examined through another multi-group APIM, whether the model in Fig 2 fitted properly for boys and girls. The unconstrained model, χ^2^ (20) = 12.826, p = .88, CFI = 1.00, RMSEA = .00, 90%CI = .000 - .030, SRMR = .021, AIC = 2377.96, was compared to a model with equality constraints between boys and girls on all structural paths. The difference in model fit, Δχ^2^ (18) = 37.818, p < .001, and the higher value of AIC = 2379.77, indicated that constraining the structural paths to be equal for boys and girls resulted in a worsening in the model fit. Given the exploratory nature of the analyses examining the moderating role of adolescent’s gender through a parental dyadic model, we examined the modification indices and two paths were selectively released, until good model fit was achieved. These paths included the stability coefficient for T1 to T3 antisocial behaviors, which was higher for girls, and the partner effect from T1 maternal PC to T2 paternal PC, which was significant only for boys. After freeing these two paths, the model fit the data significantly better, Δχ^2^ (2) = 16.626, p < .001, AIC = 2367.15 than the initial one with paths constrained to be equal, and we retained it as the final model.

## Discussion

Maternal and paternal parenting in two-parent families fundamentally occurs at a dyadic level. However, very few studies have tested the dyadic contribution of mothers’ and fathers’ PC on adolescents’ well-being. The present study examined the longitudinal mutual associations between maternal and paternal use of PC in a dyadic framework and the effect of these associations on adolescents’ adjustment (i.e. *antisocial behaviors* and *anxiety/depression symptoms*). To contribute to the ongoing debate about whether the link between PC and adolescents’ functioning is similar across cultures, the hypothesized associations were also tested cross-culturally in families from three countries: Italy, USA, and Colombia. Furthermore, we examined whether associations between PC and adolescents’ adjustment varied by parents’ and adolescents’ gender.

Results showed that, invariantly across countries, mothers and fathers influenced each other in their use of PC over time and that maternal and paternal PC was differently associated with adolescents’ antisocial behaviors and anxiety/depressive symptoms. No parental gender differences were found in the associations, but two associations were found to differ between adolescent boys and girls.

### A dyadic longitudinal model for maternal and paternal pc and youth-reported anxiety/depression and antisocial behaviors

To address the first aim, the potential longitudinal dyadic associations between maternal and paternal use of PC were examined in the present study using the Actor–Partner Interdependence Model (APIM) to account for the interdependence between partners and to test for possible actor and partner effects [63]. We found evidence of moderate stability (*actor effects*) in maternal and paternal PC over 2 years, suggesting that in family life there was continuity in the use of parental PC for both mothers and fathers when their adolescents were 13 and 14 years old. We also found evidence of dyadic associations across time between maternal and paternal PC. Significant partner effects were found for both mothers and fathers, meaning that those parents who reported more use of PC with their adolescents tended to have partners that use more PC one year later. This finding is consistent with theory and studies that found that associations among mothers’ and fathers’ PC and autonomy granting were moderately high (e.g., [78]), suggesting that mothers and fathers engage in relatively similar parenting behaviors during interactions with their child as a desire to present their children with consistent expectations, but the results showed also that parents are influenced by the behavior of their partner during family interactions [18, 79].

Parental PC was found to be predictive of both *antisocial behaviors* and *anxiety/depression symptoms* when adolescents were 15 years old, with some differences between mothers and fathers. Specifically, maternal PC was positively associated only with adolescents reported *antisocial behaviors*, whereas paternal PC was associated with anxious-depressed symptoms, but, contrary to our expectations, this association was negative, suggesting that adolescents whose fathers tended to use more psychologically controlling strategies reported fewer internalizing symptoms.

This was a surprising finding, as it is generally found that PC is positively linked to children’s maladjustment [1, 67, 78]. Reasons that our findings differed from those of previous studies may have to do with the different role mothers and fathers are perceived to play in the family interactions by their children [80]. Some authors suggested that when examining PC and its features across cultures, researchers should focus more on the *perceived* PC rather than on the actual behaviors (e.g., [46]). Accordingly, but tentatively, the positive association between paternal PC and adolescents’ anxiety-depression may indicate a more positive view of paternal controlling strategies perceived by the adolescents as a way to engage more in their life and, consequently, being more present in the parent-child relationships (e.g. [31]).

This explanation may be justified by the vast literature supporting the idea of fathers being less involved than mothers in the family dynamics, devoting less time than women in parenting, and being perceived as less psychologically controlling than mothers [27, 29, 81]. It is possible that families in which fathers are (highly) involved are characterized by different effects of PC in ways that are related to parental autonomy granting [82, 83]. Furthermore, the fact that especially paternal, and not maternal, PC had a relevant role, even if in the opposite of expected direction, on adolescents’ internalizing symptoms, is in line with the work of Lansford and coll. [20] that reported that fathers’ parenting, but not mothers’ parenting, was a unique predictor of adolescents’ internalizing problems and externalizing problems. Similarly, Verhoeven and coll. [84] found that paternal over control was more important than maternal over control in adolescents’ anxiety levels and that this association was stronger for older than for younger adolescents (<15 years of age). The authors also found a controversial result, namely that higher levels of paternal autonomy granting—over and above maternal autonomy granting—were related to higher levels of anxiety, for elementary school-aged children (age >10). For older adolescents a significant positive association between paternal autonomy-granting and anxiety was found, but its strength was marginal. Taken together, these findings call for future studies to further explore and examine the extent to which paternal involvement and perceived PC might explain mothers’ and fathers’ different effects on adolescents’ adjustment.

Findings showing that maternal PC was associated with antisocial behaviors, but not internalizing behaviors, were consistent with other studies that showed, when considering both maternal and paternal reports, that maternal PC was not related to adolescents’ internalizing symptoms and /or it was only related to externalizing features of adolescents’ behaviors (e.g., [11, 85]). This line of research suggested that when youth does not perceive support for their desire for greater independence, they may be more likely to respond to maternal PC by engaging in defiant and noncompliant behavior to exert their autonomy. By contrast, our findings were different from those found by other authors that found maternal PC to be associated with higher levels of adolescents’ depressive symptoms over and above paternal PC (e.g., [78]) and those showing that maternal and paternal PC are associated with both internalizing and externalizing symptoms [78, 85].

In addition to direct effects, our aim was also to test for longitudinal indirect actor and partner associations linking parents’ PC and adolescents’ adjustment through the partners’ parenting. Results showed evidence of one indirect association suggesting longitudinal effects of one’s own and partners’ levels of PC in shaping the development of adolescent adjustment. Specifically, a positive longitudinal indirect association emerged linking paternal PC and adolescents’ antisocial behaviors through maternal PC, meaning that the effect of paternal PC on adolescents’ behavior problems was also determined by the influence that mothers’ parental strategies had on those used by their partners. This longitudinal indirect actor association with adolescents’ adjustment trough partners’ levels of parenting was very small in magnitude and the findings need to be taken with caution in their interpretation. However, it suggested the long-lasting implications of mothers’ and fathers’ reciprocal influences on adolescents’ antisocial behaviors. These patterns suggested that when considering the effects of parental PC on adolescents’ adjustment, mothers’ and fathers’ perceptions play a unique role in shaping family dynamics, by not only serving as a direct influence on their own subsequent use of PC, but also conditioning, indirectly via affecting their partner’s use of PC, adolescents’ long-term adjustment. According to Granic and Patterson’s [86] conceptualization of “feedback family processes”, inadequate parenting practices direct children to escalating antisocial behavior that, in turn, elicits increased negative parenting practices. Yet problems or vulnerabilities originating in the parental dyad (e.g., lack of resources, negative communication, parenting-related stress) may also start such a vicious cycle. For example, it is likely that those parents experiencing difficulties in dealing with the growing desire for autonomy of their offspring will engage in psychologically controlling behaviors, which would then influence their partner’s feeling about adolescents’ autonomy and reinforce or contrast the tendency to elect PC as the most effective strategy to maintain family homeostasis [9]. Prior research has noted the way that negativity can escalate between partners into a dynamic referred to as “reciprocity” [87]. Based on the small effects found in the present study, these results support the urge for researchers (e.g., [63]) to take a couple-based (dyadic) approach in both basic research on family functioning and practical intervention efforts with couples and families.

Adolescence is especially a time of vulnerability for antisocial behavior [88], and the results of the present study add to the growing evidence of the importance of maternal PC for externalizing problems [28]. Future studies should also continue to explore potential parental gender differences and the ways in which identification with the parenting role or involvement in the relationship may influence the associations between parental PC and adolescents’ adjustment.

Understanding parental differential contributions to adolescent behavior problems will help to identify focal points for intervention. These results also carry implications for the question of whether considering maternal and paternal use of different manipulative strategies may provide differential and unique contributions to adolescents’ adjustment over time (e.g., [89]). We considered the differences in the associations between maternal and paternal strategies as evidence of the importance of conceptualizing a model in which mothers’ and fathers’ dyadic influences are considered.

### Multigroup cross-cultural comparison

The second aim of the study was to test the generalizability of the hypothesized associations across three countries: Italy, Colombia, and the USA. From the multigroup cross-cultural comparison, we found evidence of cross-cultural invariance of dyadic associations across time between maternal and paternal PC. Significant and positive actor and partner effects were found for Italian, Colombian, and US mothers and fathers, meaning that in the three countries, parents not only tend to be consistent in their use of PC with their adolescents over time (*actor effects*), but they also influence their partners’ use of PC one year later (*partner effects*) (e.g. [18]). Very limited prior empirical work examined in a cross-cultural framework whether maternal and paternal PC have a reciprocal influence over time such that one parent’s tendency to engage in a manipulative parental strategy might in turn influence their partner’s parenting as well (e.g., [19]). Among the few studies that attempted to address this hypothesis, a study on the effect of PC and internalizing distress in Italian emerging adults hypothesized that interdependence among family members could explain the potential differences in the effects of PC among countries [54]. The authors hypothesized that PC in Italy may have a less negative effect on individual adjustment compared to other Western countries like the USA because of the relevance of interdependence and loyalty values in Mediterranean culture [52, 53]. Interdependence and relatedness characterize relationships within families and could account for the presence of reciprocal effects within the parental dyad found in the present study. The use of PC that highlights the importance of family bonds and intergenerational loyalty, by introducing autonomy-related processes, might be characterized by a higher agreement between mothers and fathers in collectivist countries (i.e. Italy and Colombia) compared to countries where independence is considered more important [90]. However, the cross-cultural invariance found in the present study, in line with the evidence found by Costa and colleagues, suggested no difference among the three countries in which both interdependence (i.e. significant partner effects) and associations with maladjustment were found.

Also, cultural beliefs likely provide a different context for thinking about the implications of paternal PC for adolescents’ adjustment. For example, an adolescent who highly identifies with the Latinx culture may view a father’s controlling style as culturally normative as a function of *familismo* or *machismo*, interpreting it as signs of love and concern [91, 92], even if studies conducted on Latinx samples showed that this is especially the case for behavioral control (e.g., [93, 94]). The present study’s lack of an acculturation and/or ethnic identity measure can certainly be viewed as a limitation, and future studies are needed to test these hypotheses.

### The moderating role of parents’ and adolescents’ gender

Our third aim included the tests of gender differences in parental actor and partner effects. Our findings showed no significant differences between mothers’ and fathers’ actor and partner effects, supporting the idea that parents that tend to behave similarly in their interactions with their children are also more likely to influence their partners in the same direction (e.g., [95]). Also, according to Luo & Klohnen [96], parents tend to influence each other and become more similar over time, which may promote adaptive family functioning when it reinforces positive practices [97].

Regarding adolescents’ gender, the comparison between girls and boys showed a difference in the hypothesized associations. Specifically, beyond the higher stability of girls’ antisocial behaviors compared to boys, the maternal partner effect from T1 to T2 was significantly stronger for boys. Taken with caution, this finding suggested that mothers’ use of PC influenced fathers’ PC one year later, especially when their controlling strategies were used with boys. Interestingly, and in line with previous studies (e.g., [43]), the results showed that the associations among maternal and paternal PC and problematic outcomes did not vary by adolescent gender. However, while effects on adjustment might remain similar, the underlying processes might vary for boys and girls as a function of the dyadic influences mothers and father exert on each other. If confirmed by future studies, this finding might contribute to the recent research on differential effects of PC when comparing same to opposite-gender parent-child dyads, by suggesting the relevance of adopting a dyadic framework when testing the effects on adolescents’ adjustment.

### Limitations and strengths

There are several limitations associated with the current study. First, this study relied on self-reports of perceived parenting and adjustment rather than including observations, although we included mothers’, fathers’, and adolescents’ reports to minimize same-source biases. Future studies might include both perceived and enacted parenting to better explore whether it is the perceived behaviors, observed behaviors, or discrepancy in these assessments of parent-child perception that is most predictive of adolescents’ adjustment (e.g., [43]). However, it was a specific aim of the study to focus on parental perception that has mostly been overlooked in research [10] and examining the dyadic dynamic among mothers and fathers.

Second, the unexpected negative effects found between paternal PC and adolescents’ internalizing symptoms need to be taken with caution given the absence of previous studies explicitly supporting this finding. Adolescents’ perceived PC and father-adolescent relationship quality tested in a dyadic framework may play a role in explaining such associations. Thus, further studies are required to test this hypothesis and further investigate this intriguing result. Also, the reliability of the antisocial behavior scale was somewhat lower as compared to the other scales. Tentatively, compared to the other scales, the low variance of some items (e.g. “*I steal things from places other than home”)* might have influenced the reliability score. Conceptually, even if it is beyond the purpose of the present study, future studies might consider to revising the construct, by, for instance, separating aggressive from non-aggressive delinquent behaviours as suggested by some studies conducted on delinquent behaviours in a cross-cultural framework [98].

Third, the association between adolescents’ adjustment and parental PC is clearly bidirectional and the relationship usually precedes parent–child relations (e.g., [2]). Being the main rationale for the present study, we focused on mother and father’s dyadic influences, implementing a specific analytical model to test them (i.e. APIM); thus, the effect of adolescent’s adjustment on parenting was not examined. However, when testing our dyadic hypotheses, we controlled from previous levels of adolescents’ antisocial and anxiety-depressive symptoms on later adjustment in order to take into account previous level of adolescents’ negative adjustment.

Finally, although the current study assessed families three times across three years, which allowed us to capture dyadic and familial dynamics over fairly long periods of time, our data cannot capture short-term associations between maternal and paternal PC. Considering that parental and couple interactions occur on a daily basis [97, 99] future studies might use daily diary methods to further explore the reciprocity between parental PC and adolescents’ adjustment across shorter periods of time.

Overall, we encourage to take these findings with caution, given the exploratory nature of the present study. A main aim of the present study was to use a dyadic, cross-cultural approach to examine mothers’ and father’s influences over time, however, current findings need further support and replication by future studies.

Despite these limitations, the present study contributes to the understanding of parental PC in several ways. First, we included both parents in the model so results and conclusion are not based solely on mothers’ contributions, overlooking paternal roles in family dynamics [89]. Second, we adopted a longitudinal dyadic approach, by considering dyads and mutual associations as the most reliable proxy of family dynamics [100, 101]. Third, including adolescents’ adjustment both in terms of internalizing and externalizing behaviors adds to the growing evidence of PC as an important predictor of both dimensions rather than solely internalizing [1, 4, 10, 64]. Fourth, the cross-cultural design of the present study and the findings of invariance of the associations provide further evidence of the generalizability of the effects of PC on family dynamics and adolescents’ adjustment across diverse cultural contexts [13, 48, 102].

Understanding the distinctiveness of maternal and paternal parenting functioning, as well as the reciprocal influences between the two, has important implications for prevention efforts in improving family functioning and child development. Professionals working in education and intervention programs hoping to enhance and/or maintain parental quality, need to be aware of the importance of including both actors in parenting dynamics since paternal PC might have effects on children’s development over and beyond mothers’ contribution, and sometimes in an unexpected direction. Accordingly, both parents should be made aware of the reciprocal influences they exert on each other in maintaining negative parental practices, as PC, and its detrimental effects on adolescents’ adjustment. Individual and/or couple-based interventions with parents should promote autonomy-supportive parenting in opposition to psychologically controlling practices. Autonomy-supportive parents are able to value the adolescents’ perspective and allow them to explore and build their own identity (e.g., [6]). The exploratory findings from the present study may suggest that, when promoting autonomy-supportive parenting behaviors, it might be beneficial to target the specific roles and influences mothers and father might exert on each other’s. As such, maternal PC- which has been receiving far more attention in the literature- should be considered in light of the influences that paternal use of PC has on mothers’ parenting. Further research is however needed to investigate this line of reasoning in greater detail, as well as to clarify its implementation in the clinical framework.

Our analyses showed that PC is similarly associated with adolescents’ well-being across diverse cultures (Italy, Colombia, and the United States). The role of cultural orientation in the dynamics of PC was advanced by including mothers’ and fathers’ reciprocal associations, suggesting how studies on PC across cultures might rely on parents’ experiences of their own and their partners’ parental choices. On one hand, the present study showed that the dyadic processes were invariant across the three countries, meaning that dyadic interplays between parents might be always expected with Italian, Colombian, and US families; on the other hand, we found different effects of PC on adolescents’ adjustment suggesting there are distinguishable forms of PC that may have different relevance across cultures. Working with Colombian couples and families may require higher sensitivity to the meaning of PC, given the importance of values of *familismo* and *respeto* for family relationships. In these families, control often has been found to be interpreted as a way of teaching children to behave consistent with the family’s values [48, 103]. Similarly, when working with Italian couples and families, the importance of family relationships in the Mediterranean culture and factors such as prolonged coresidence of youths with their parents (e.g., [53]) should be taken into account when focusing on PC-related processes and their autonomy-related effects [52]. Finally, in more individualistic contexts such as the USA, parents who are psychologically controlling were found to hold more negative perceptions about their children with important implications in both parental and parent-youth dyadic processes [104, 105]. Professionals and family researchers–including those working with families from different cultural backgrounds—should also be aware of these cultural features when focusing on the reciprocal influence mothers and fathers exert on each other and on adolescents’ adjustment.

## Conclusions

The results support theoretical frameworks showing the effects of PC are generalizable across cultures [99, 106]. Given our findings of partner effects in the longitudinal associations in parental PC, it is important to take a dyadic cross-cultural perspective when examining relationship processes. Overall, the results of the present study suggested that practices involving conditional love, guilt induction, and love withdrawal are deeply embedded in family dyadic interactions over time and across cultures.

## Supporting information

S1 Table Multigroup APIMs by country, parent and adolescents’ gender. (DOCX)

S1 Data Data included in the paper. (XLS)
