# Supplementary material for: Maternal and paternal psychological control and adolescents’ negative adjustment: A dyadic longitudinal study in three countries
Source: PLoS One. 2021 May 14;16(5):e0251437. doi: 10.1371/journal.pone.0251437 (PMC8121295; doi:10.1371/journal.pone.0251437)
Supplement: S1 Table — (DOCX) [file pone.0251437.s001.docx]

**S1 Table. Multigroup APIMs by country, parent and adolescents’ gender.**

|  | **Multi-Group APIM for Study Sites** | | | | | | | | | | | | |
| --- | --- | --- | --- | --- | --- | --- | --- | --- | --- | --- | --- | --- | --- |
|  | | χ ^2^ | *df* | CFI | AIC | RMSEA  (95% C.I.) | SRMR | Model Comparison | χ^2^ _diff_ | ∆*df* | *p* |  |  |
| Model 1. Unconstrained Model | | 34.924 | 30 | .992 | 2344.60 | .036 (.000 - .080) | .038 |  |  |  |  |  |  |
| Model 2. Full Constrained Model | | 76.261 | 66 | .984 | 2313.69 | .035 (.000 - .066) | .091 | 2 vs 1 | 41.337 | 36 | *p=.24* |  |  |
|  | |  |  |  |  |  |  |  |  |  |  |  |  |
|  | **Multi-Group APIM for Mothers’ and fathers’ Actor and Partner effects** | | | | | | | | | | | | |
|  | | χ ^2^ | *df* | CFI | AIC | RMSEA  (95% C.I.) | SRMR | Model Comparison | χ^2^ _diff_ | ∆*df* | *p* |  |  |
| Model 1. Unconstrained Model | | 9.189 | 10 | 1.00 | 2428.61 | .000 (.000 -.053) | .018 |  |  |  |  |  |  |
| Model 2. Full Constrained Model | | 10.515 | 12 | 1.00 | 2334.24 | .000 (.000 - .047) | .026 | 2 vs 1 | 1.326 | 2 | *p=.51* |  |  |
|  | |  |  |  |  |  |  |  |  |  |  |  |  |
|  | **Multi-Group APIM for Adolescents’ Gender** | | | | | | | | | | | | |
|  | | χ ^2^ | *df* | CFI | AIC | RMSEA  (95% C.I.) | SRMR | Model Comparison | χ^2^ _diff_ | ∆*df* | *p* |  |  |
| Model 1. Unconstrained Model | | 12.826 | 20 | 1.00 | 2377.96 | .000 (.000 -.030) | .021 |  |  |  |  |  |  |
| Model 2. Full Constrained Model | | 50.644 | 38 | .983 | 2379.77 | .042 (.000 - .070) | .079 | 2 vs 1 | 37.818 | 18 | *p<.001* |  |  |
| Model 3. Partial Constrained Model | | 34.018 | 36 | 1.00 | 2367.15 | .000 (.000 - .048) | .056 | 3 vs 2 | 16.626 | 2 | *p<.001* |  |  |
